# Isolation and Molecular Identification and Antimicrobial Susceptibility of *Providencia* spp. from Raw Cow's Milk in Baghdad, Iraq

**DOI:** 10.1155/2020/8874747

**Published:** 2020-11-19

**Authors:** Nagham Mohammed Ayyal Al‐Gburi

**Affiliations:** Zoonotic Diseases Unite, College of Veterinary Medicine, Baghdad University, Baghdad, Iraq

## Abstract

A total of sixty raw milk samples were collected from (street vendors and shops) from Baghdad city, Iraq. The samples were inoculated into peptone water and, then, subcultured onto MacConkey agar and Blood agar. Identification of isolates was confirmed by microscopic examination, cultural characteristic, biochemical tests, Vitek (VITEK®2 system), and Biolog GN substrate reactions followed by 16S rRNA and specific genes sequencing. Of 60 raw cow's milk samples, *Providencia* spp. were identified only in 4 samples (6.67%) and *P. rettgeri* was the most common, 2/4 (50%), followed by *P. stuartii* and *P. vermicola*, 1/4 (25%). Antimicrobial susceptibility tests were conducted against ten antibiotics by the disc diffusion method. All *Providencia* isolates showed multidrug resistance (MDR), and the absolute resistant was 100% to tetracycline, erythromycin, and doxycycline and 50% against ampicillin\sulbactam and amoxicillin/clavulanic acid. They were highly susceptible (100%) to trimethoprim, imipenem, and chloramphenicol. These findings indicate that milk might be contaminated with *Providencia* spp. leading to transmission to humans causing poisoning, diarrhea, and other infections. This is the first study of isolated *Providencia* spp. from raw cow's milk.

## 1. Introduction

Before 2005, the genus *Providencia* was including six species; after that, a new species was identified to become nine species which are *P. alcalifaciens*, *P. rustigianii*, *P. stuartii*, *P. rettgeri*, *P. friedericiana*, *P. heimbachae*, *P. vermicola*, *P. sneebia*, and *P. thailandensis* [1–3]. The *Providencia* spp. are urease-producing Gram-negative, belonging to the family Enterobacteriaceae. Although these species are present as normal flora in the human intestinal tract, they are opportunistic pathogens, especially in immunocompromised people causing traveler's diarrhea, gastroenteritis and infection of the urinary tract and endocardium, sepsis in neonatal, and ocular inflammations [4–9]. Animals such as cattle, sheep, insects, worms, cats, birds, dogs, guinea pigs, and reptiles are reservoirs to *Providencia* spp., as well as this bacterium present in environment such as water (river, cows, and waste) [10–14]. These may explain the isolation of them from different food and food products [9, 15–17].

The laboratory identification of *Providencia* spp. is depending on culturing and biochemical characteristics. *Providencia* spp. grow in enteric agars such as MacConkey, *Salmonella*-*Shigella* (SS), Eosin Methylene Blue (EMB), and Hektoen Enteric (HE), and selective agars are Simmons Citrate, Tergitol, and HardyCHROM™ UTI [18, 19]. The commercial identification kits currently available include the Analytical Profile Index (API20) Esystem, Vitek GNI and GNI1 cards, and Microscan Rapid Neg ID3panel [20–23] in addition to the molecular identification using 16S rRNA and specific species genes [10, 24, 25].

*Providencia* spp. reported resistant to antimicrobials and multidrug resistance (MDR), and both *P. stuartii* and *P. rettgeri* reported resistant against many antimicrobial drugs. *Providencia* isolates were investigated to be MDR (75%) [26, 27]. *P. rettgeri* isolates are found highly resistant against several antimicrobials such as gentamicin, imipenem, polymyxin, tetracycline, nitrofurantoin vancomycin, bacitracin, erythromycin, novobiocin, rifampin, and colistin [28, 29]. *P. vermicola* has reported 100%resistance to ampicillin, kanamycin, nalidixic acid, and neomycin [25]. Carbapenem-resistant *P. stuartii* and *P. rettgeri* were reported; therefore, the treatment has been a problem because MDR is a significant public health challenge [30–32]. Milk has been considered the most nutritious and balanced food being rich in essentials that are important for humans [33].

Milk is considered as a perfect environment for the growth and survival of many microorganisms that are a threat to the public health [34–36]. Contamination of milk with microorganisms occurs via a variety of environmental sources, including soils, water, moisture content, milking apparatus, and surrounding air condition [33, 37, 38]. There are rare investigations of *Providencia* spp. in raw cow's milk, especially in Iraq. It has been found that *Providencia* spp. is one of the bacteria that were isolated from subclinical mastitis [39, 40]. This study was conducted to detect the presence of *Providencia* spp. in raw cow's milk and determine the antimicrobial susceptibility to antibiotics.

## 2. Materials and Methods

### 2.1. Isolation and Morphological Identification

Sixty raw milk samples were collected from vendors and shops in Baghdad city from March to June 2019. Ten ml of milk samples was taken and inoculated into peptone water, incubated at 37°C for 24 hrs, then subcultured on MacConkey agar and Blood agar (HiMedia, India), and incubated at 37°C for 24 hrs. The suspected *Providencia* spp. colonies which appear as pale colonies, lactose nonfermenter on MacConkey agar were picked, and conventional biochemical tests were applied including urease, phenylalanine, and triple sugar iron (TSI). They were further identified using the VITEK®2 system and Biolog GN substrate (Biomerieux, France) reactions for more differentiation according to [1, 41].

### 2.2. Molecular Identification

Molecular identification was applied on three isolates to confirm and differentiate between them. DNA was extracted from isolates growth using the Wizard Genomic DNA Purification Kit protocol (Promega, USA). PCR amplification of bacterial 16S rRNA was applied with 27 forward primer AGAGTTTGATCCTGGCTCAG and 1492 reverse primer TACGGTTACCTTGTTACGACTT 1, 300 bp. The PCR reaction mixture final volume of 25 *μ*l contains PCRpremix 12.5 *μ*l, forward primer 1 *μ*l, reverse primer1 *μ*l, nuclease free water 8.5 *μ*l, and DNA 2 *μ*l. The PCR scheme performed was as follows: 95°C for 5 min/1 cycle; 95°C for 30 sec/30 cycles; and 60°C for 45 sec/30 cycles and extension at 72°C for 1 min/30 cycles and final extension 72°C for 7 min/1 cycle [42]. After amplification, 1% agarose gel electrophoresis was applied to confirm the presence of amplification. Then, the PCR products were purified and sent to be sequenced.

Provi_forward primer CGCATAATCTCTTAGGAGCAAA and Provi_reverse primer ATGAATCACAAAGTGGTAAGCG (size 1306 bp) were used to detect both *P. rettgeri*/*P. vermicola*, Provi_foward primer and P_Vermi_reverse primer (AAGGAGR (A/G) TGATCCAGCCGCAG) (size 1366 bp) were used to detect if the isolates are *P. vermicola* or not. The PCR reaction mixture volume of 20 *μ*l included PCRpremix 10 *μ*l, nuclease free water 6 *μ*l, forward and reverse primer 1 *μ*l to each and DNA 2 *μ*l. The PCR scheme performed was as follows: initial denaturation at 95°C for 5 min, denaturation at 95°C for 30 sec, annealing at 62°C for 30 sec (68°C for 30 sec for Provi_foward primer and P_Vermi_reverse primer), extension at 72°C for 1 min, and final extension 72°C for 7 min [25].

After amplification, 1% agarose gel electrophoresis was applied to confirm the presence of amplification, then PCR products were purified and sent to sequenced, and the results were analyzed using genius software and compared to known sequences in the GenBank and Sepsitest BLAST databases.

### 2.3. Detected Antimicrobial Susceptibility

Susceptibility against antimicrobial drugs was determined by disk diffusion protocol using Mueller-Hinton (MH) agar (Oxoid, UK). The inhibitory zones around these antimicrobial discs were measured using a millimeter (mm) unit utilizing a metric ruler, and the results were read [43, 44]. Ten antibiotic disks (Merseyside, U.K.) used included amoxicillin 20 *µ*g, clavulanic acid 10 *µ*g (AUG 30C), trimethoprim (TM, 15 *µ*g), ampicillin10 *µ*g\ sulbactam 10 *µ*g (SAM, 20 C), tetracycline (*T*, 30 *µ*g), erythromycin (*E*, 10 *µ*g), cefixime (CFM 5 µg), doxycycline (DXT, 30 µg), imipenem (IPM, 10 µg), chloramphenicol (*C* 30 *µ*g), and streptomycin (*S* 25 *µ*g). Multidrug resistance (MDR) was detected according to the work of Magiorakos et al. [45]. The isolates resistant against three or more separate antimicrobial classes are considered as MDR. The multiple antibiotics resistance (MAR) index was calculated by dividing (a): the number of antimicrobial drugs resistant of isolate by (b): the total number of antimicrobial drugs, where the same isolate which exposed the results more than 0.2 was considered high risk [46].

## 3. Results

### 3.1. Characterization and Molecular Identification of *Providencia* Species

*Providencia* species were identified in 4 (6.67%), P. *rettgeri* were the most dominant species, 2/4 (50%), and *P. stuartii* and *P. vermicola* were 1/4 (25%). The isolates were Gram-negative coccobacilli. On MacConkey agar, lactose nonferment colonies are circular with entire edges, shining, smooth, slim, and convex, *P. vermicola* showed a dense brownish center and hyaline periphery colonies, and the isolate on blood agar are nonheamolysis. Biochemically, it is motile, negative reactions for oxidase, positive for catalase and tryptophan deaminase, on TSI, gives alkaline/acid (pink/yellow) reaction without H_2_S and gases production, the isolates were suspected of *Providencia* spp., and by using the VITEK®2 system, reactions give the positive for three isolates of *P. rettgeri* and one isolate was *P*. *stuartii*.

**Figure 1:**
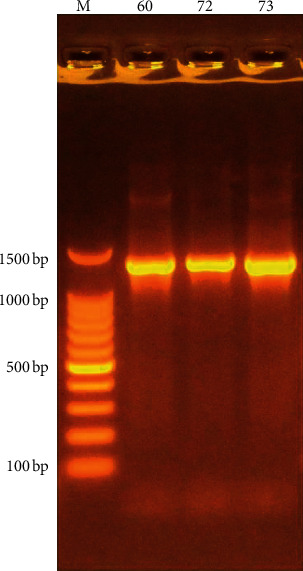
The amplification of species-specific primer1306 bp for *P. rettgeri*/*P. vermicola* genes of isolates, fractionated on agarose gel electrophoresis 1% stained with Eth. Br, M 100 bp DNA marker (60, 72, and 73 refer to the no. of *Providencia* species isolates).

*P. rettgeri* isolates (unfortunately, *P*. *stuartii* isolate died before completing molecular identification) were sent to be sequenced and analyzed for similarity using a database at the NCBI. The partial gene sequence of 16S rRNA established that these isolates had high similarity between *P. rettgeri/P. vermicola* in both databases, when using Provi_foward and Provi_reverse (detected both *P. rettgeri* and *P. vermicola*), and a band of approximately 1306 bp was observed on the agarose gel (Figure 1). The results of the sequence were as follows: 1^st^ isolate was 99.91% similar with *P. rettgeri,* the 2^nd^ gave 100% similarity to *P. rettgeri/P. vermicola*, while the 3^rd^ isolate was 99.81% similar to *P. vermicola/P. rettgeri* in the Gen bank database and in the Sepsi test BLAST database; similarity were as follows: the 1^st^ was *P. vermicola/P. rettgeri*, 99.5%; the 2^nd^ was *P. vermicola/P. rettgeri*, 99.9%, while the 3^rd^ was *P. vermicola*, 99.8%, and when using Provi_forward and P_Vermi_reverse primer (detected only *P. vermicola*), the 1^st^ and 2^nd^ isolates give no bands, that is, these two isolates were *P. rettgeri,* while the 3^rd^ one gives two bands, which were not of the same expected size of 1366 bp; therefore, this isolate was more differentiated from *P. rettgeri* based on Biolog GN substrate reactions for more biochemical tests, and the sequencing result gives that the 3^rd^ isolate was *P. vermicola*, 99.8%; thus, the three isolates were deposited in GenBank with accession nos : MT032351.1, MT032352.1, and MT032359.

### 3.2. Antimicrobial Susceptibility

As total, all *Providencia* isolates showed absolute resistance (100%) against tetracycline, erythromycin, and doxycycline, 50% to Amoxicillin\clavulanic acid and ampicillin\sulbactam, and 25% to cefixime. *P. vermicola* was resistant 100% to amoxicillin 20 *μ*g\clavulanic acid and ampicillin\sulbactam compared with *P. rettgeri* which was 50% and *P. stuartii* was 0%. The resistance against streptomycin reported 50% in *P. rettgeri*. Also, resistance to cefixime was 100% against *P. vermicola,* while 0% (sensitive) in both *P. rettgeri* and *P. stuartii*. All isolates were 0% resistant (100% sensitivity) to trimethoprem, imipenem, and chloramphenicol. The results also revealed that these isolates were MDR. MAR index values were 0.6 in *P. rettgeri* and *P. vermicola* and 0.3 in *P. stuartii* that reveals all isolates were high risk (Table 1).

## 4. Discussion

Infections with *Providencia* spp. including *P. rettgeri* and *P. stuartii* which cause food poisoning, diarrhea, and UTI have been increased in the world, especially in developed and developing countries [9, 32, 47–51]. *P. vermicola* was investigated for the first time in infective nematodes, later from a diseased fresh water fish and from an acute watery diarrhea patient [1, 25, 52]. Therefore, in the present study, we investigate for *Providencia* spp. in raw milk collected from shops and vendors in Baghdad city. Our results indicate that raw milk is contaminated with *P. rettgeri*, *P. stuartii*, and *P. vermicola. Providencia* spp. including *P. rettgeri* and *P. stuartii* were isolated from food [9, 15–17, 53, 54]. In Iraq, out of bacterial content of fish gut, *P. rettgeri* reported 1/50 [55]. This is the first report of isolated *Providencia* from raw milk in Iraq; there is a study in Kenya, where 0.6% *Providencia* spp. was isolated from the milk of goats with subclinical mastitis, and 2% *P. stuartii* and *P. alcalifacians* were isolated from clinical healthy cows (subclinical mastitis) in Algeria [39, 40]. This study is also the first isolating *P. vermicola* from a food source such as milk. Milk may be contaminated by the environment such as soil, water, feces of the carrier or infected cattle, unhygienic conditions during milking, or used contaminated containers.

Phylogenetically, the family of Enterobacteriaceae has a highest similarity (16S rRNA gene sequence similarity), particularly with the members of genus *Providencia* (>98.1%), and a higher similarity 99.5% was found between *P. rettgeri* and *P. vermicola* [1]. In agreement with that found in the present study, the similarity was ranging from 99.81 to 99.9% even in used species-specific gene. In contrast, the primer Provi_foward and P_Vermi_revers primers confirmed that the two isolates were *P. rettgeri* (no bands), but the 3^rd^ one gives more one band in suspected *P. vermicola*. For this reason and because of the fact that the VITEK®2 system does not contain automated identification of *P. vermicola* in the list of card Gram negative, we used Biolog GN substrate reactions for more biochemical tests to differentiate *P. vermicola* from *P. rettgeri* such as urease, erythritol and 2-ketogluconate, L-arabinose, D-glucosaminic acid, and D-glucuronic acid reactions and depending on the cultural characterization according to [1, 41]. In addition to the sequence, results confirmed that this isolate was *P. vermicola*.

In the current study, the results showed multiple resistance to antibiotics with high risk, these results were similar to other research studies, and resistance against multiple antimicrobials including tetracycline, ampicillin, and streptomycin were recorded in *Providencia* isolates in farm animals [56]. On the other hand, MDR *P. rettgeri* from UTI patients recorded high resistance to amikacin, aztreonam, cephalosporins, ciprofloxacin, ertapenem, and meropenem [41]. MDR (43%) showed in *Providencia* spp. including *P. stuartii* and *P. rettgeri* isolated from retail meats, and most of the isolates were resistant (91%) against tetracycline, ampicillin (69%), and streptomycin (49%) [54].

In addition, the results are similar with some differences in percentage of resistance to the study in Iraq; *P. alcalifaciens and P. rettgeri* isolated from clinical sputum and wastewater showed a high resistance against nitrofurantoin (100%), ampicillin (94.4%), amoxicillin/clavulanic acid (72.2%), and ampicillin/sulbactam and tetracycline (38.8% and 33.3%), respectively, while resistance to imipenem and cefotaxime were 5.6% each, and the most effective antibiotics were 100% resistant to norfloxacin, chloramphenicol, and cefixim and 88.9% resistant to trimethoprim recorded by the authors in [14] which are accordance with our results. High drug resistance against chloramphenicol, trimethoprim, and tetracycline and susceptibility to streptomycin of *P. vermicola* were recorded in diarrheal patients [25]. Despite MDR and the risk of the *Providencia* spp. isolates, all the isolates were susceptible to important antimicrobials which are used clinically such as imipenem, trimethoprim, and chloramphenicol in the present study.

## 5. Conclusions

Our results showed that raw milk is a potential source of *Providencia* spp. that may lead to infection in humans and risk to public health, especially the bacteria found as MDR. The present *Providencia* spp. in milk may be attributed to contamination after milking from the environment such as animal feces, or it is source from subclinical mastitis cow.

## 6. Recommendations

More studies should be conducted for more identification of this microorganism in food and its products in Iraq. Conventional methods and commercial kits beside molecular techniques should be used to identify *Providencia* spp. in level species.

## Figures and Tables

**Table 1:** Antimicrobial susceptibility results of *Providencia* spp. isolated from milk.

Antibiotic	Resistance (%)			
	*P. rettgeri* [2]	*P. vermicola* [1]	*P. stuartii* [1]	Total
Amoxicillin/clavulanic acid	50	100	0	50
Streptomycin	50	0	0	0
Trimethoprim	0	0	0	0
Ampicillin\sulbactam	50	100	0	50
Tetracycline	100	100	100	100
Erythromycin	100	100	100	100
Cefixime	0	100	0	25
Doxycycline	100	100	100	100
Imipenem	0	0	0	0
Chloramphenicol	0	0	0	0
MAR index	0.6	0.6	0.3	

## Data Availability

Data used to support the findings of this study can be obtained from the corresponding author on request.
