# Corrigendum to “Isolation and Molecular Identification and Antimicrobial Susceptibility of *Providencia* spp. from Raw Cow's Milk in Baghdad, Iraq”

**DOI:** 10.1155/2021/2954176

**Published:** 2021-01-28

**Authors:** Nagham Mohammed Al‐Gburi

**Affiliations:** Zoonotic Diseases Unit, College of Veterinary Medicine, University of Baghdad, Baghdad, Iraq

In the article titled “Isolation and Molecular Identification and Antimicrobial Susceptibility of *Providencia* spp. from Raw Cow's Milk in Baghdad, Iraq” [1], the name of the author was given incorrectly as Nagham Mohammed Ayyal Al‐Gburi. The author's name and affiliation should be corrected as shown above.

Additionally, there was an error in the second sentence of the abstract and the second sentence of the Materials and Methods section, where the word “McConkey” should be corrected to “MacConkey.”
